# Organ-specific local cytokine release syndrome after anti-CD19 CAR-T therapy with salivary gland involvement: a case report, literature review, and a diagnostic alert for tocilizumab-associated cervical swelling

**DOI:** 10.1007/s00277-026-07013-0

**Published:** 2026-04-22

**Authors:** Roberto Maggi, Enrico Federico Ricciuti, Eugenio Galli, Federica Sorà, Antonella Fiorita, Simona Sica, Patrizia Chiusolo

**Affiliations:** 1 Hematology Division, Policlinico Universitario Agostino Gemelli, Largo Francesco Vito 1, Rome, Italy; 2 Università Cattolica del Sacro Cuore, Rome, Italy; 3 Otorhinolaryngology Division, Policlinico Universitario Agostino Gemelli, Rome, Italy

**Keywords:** CAR-T, Cytokine release syndrome, Local-CRS, Salivary gland swelling, Tocilizumab, Hypersensitivity, Airways impairment

## Abstract

Background. Cytokine release syndrome (CRS) is the most frequent toxicity after chimeric antigen receptor T-cell (CAR-T) therapy and typically presents as a systemic inflammatory syndrome. In recent years, a localized form of CRS (L-CRS), most commonly involving the craniocervical region, has been increasingly recognized. L-CRS may occur with or without overt local tumor involvement and can progress to airway impairment, requiring prompt recognition and treatment. Case presentation. We report two cases of patients with refractory diffuse large B-cell lymphoma (DLBCL) treated with anti-CD19 CAR-T therapy who developed abrupt, painful bilateral parotid and/or submandibular gland swelling (left predominance) early after infusion, following systemic CRS treated with tocilizumab. Infectious, obstructive, and autoimmune causes were excluded; ultrasound findings were compatible with inflammatory glandular changes. Given rapid progression and concern for airway patency, corticosteroids were administered with prompt clinical resolution. Literature review and diagnostic alert. We provide an overview of published L-CRS with craniocervical presentations (Table 1). Similar acute swelling temporally related to tocilizumab has been reported in other settings. In such cases, differential diagnostics of L-CRS vs. a tocilizumab-associated infusion-related/hypersensitivity reaction needs to be carried out. Conclusion. L-CRS is a clinically relevant and potentially severe complication of CAR-T therapy that may involve salivary glands even without cervical tumor burden. Clinicians should also consider drug-related reactions, particularly when cervical swelling occurs shortly after tocilizumab administration.

## Introduction

Chimeric antigen receptor T-cell (CAR-T) therapy has transformed the treatment of relapsed or refractory B-cell malignancies. Cytokine release syndrome (CRS) remains the most common toxicity, occurring in a large proportion of treated patients and classically presenting with systemic features such as fever, hypotension, and hypoxia [1].

Beyond the systemic syndrome, localized cytokine release syndrome (L-CRS) has emerged as a distinct clinical phenotype characterized by inflammatory manifestations confined to a specific anatomical compartment—most frequently the craniocervical region [2] Reports describe cervical soft-tissue edema, salivary gland swelling, and laryngeal/pharyngeal involvement, sometimes culminating in airway obstruction. Notably, L-CRS may arise even in the absence of clinically evident tumor infiltration at the affected site, suggesting mechanisms beyond a simple “on-target, on-tumor” local effect [2]. A similar but not overlapping phenomenon is the Local immune effector cell-associated toxicity syndrome (LICATS), reported when treating patients with auto-immune diseases with CAR-T: LICATS only involves organs previously affected by the respective autoimmune disease, mainly skin and kidneys, despite rarer localization have been reported [3].

Current mechanistic models propose that CRS can evolve through phases, where an initial systemic cytokine surge is followed by redistribution of activated CAR-T cells and inflammatory mediators into specific tissues, leading to compartmentalized hyperinflammation (L-CRS). This paradigm helps explaining clinical scenarios in which localized swelling occurs after systemic CRS improvement and may be incompletely controlled by Interleukin-6 (IL-6) receptor blockade alone, while responding promptly to corticosteroids [4].

IL-6 is central to CRS biology. IL-6 signaling includes “classical” signaling through membrane-bound IL-6 receptor (mIL-6R) and “trans-signaling” through soluble IL-6 receptor (sIL-6R), both converging on gp130-dependent pathways that can amplify inflammation. In advanced or tissue-compartmentalized inflammation, IL-6 receptor blockade may be insufficient, and corticosteroids are often required to rapidly reverse swelling and prevent airway compromise [4, 5]. 

Here, we report one case of salivary gland–localized CRS after anti-CD19 CAR-T therapy with rapid steroid responsiveness. A focused literature search was performed in PubMed to identify published cases of localized cytokine release syndrome (L-CRS) involving the head and neck region following CAR-T therapy. The search included articles published up to May 2025, using combinations of the terms “CAR-T”, “cytokine release syndrome”, “local CRS”, “cervical”, “laryngeal”, and “salivary gland”. Only reports describing clinical cases with localized manifestations in the craniocervical region were included. (Table 1). Finally, we present a second case in which acute submandibular swelling occurred within 1–2 h after tocilizumab administration, highlighting a potential diagnostic pitfall: not all post–CAR-T cervical swelling necessarily reflects L-CRS, and infusion-related or hypersensitivity reactions to tocilizumab should be considered when the temporal pattern is strongly suggestive. Similar acute swelling temporally associated with tocilizumab has been reported outside the hematologic setting [6–8]. 

**Table 1:** Summary of reported craniocervical manifestations of localized cytokine release syndrome after CAR-T therapy

Author	Number of cases	Site of local CRS (as reported)	Time to onset of local CRS (days from CAR-T infusion)	Local CRS occurring at a site involved by underlying disease (Yes/No)	CAR-T product
Kawase et al.	3	Bilateral salivary glands (parotid and submandibular)	Case 1. Day + 5 Case 2. Day + 4 Case 3. Day + 4	No	Mixed (liso-cel, tisa-cel)
Inoue et al.	2	Case 1: bilateral parotid and submandibular glands; Case 2: cervical region with pharyngeal/laryngeal involvement	Case 1. Day + 5 Case 2. Day + 3	No	Tisa-cel
Ko et al.	1	Bilateral salivary glands Pharynx/supraglottic region; Deep cervical soft tissues	Day + 4	No	Tisa-cel
Nakanishi et al.	1	Laryngeal region	Day + 4	No	Ide-cel
Luan et al.	1	Cervical region (neck swelling with dyspnea; no specific salivary gland or laryngeal involvement reported).	Day + 8	No	CD19-directed CAR-T (CD28 costimulation)
Jin et al.	1	Cervical lymph node enlargement (neck region)	not clearly specified	Yes	CD19-directed CAR-T
Nakamura et al.	2	Craniocervical region: subcutaneous edema, pharyngeal and epiglottic involvement	Case 1. Day + 4 Case 2. Day + 3	Case 1. No Case 2. Yes	Tisa-cel
Shima et al.	1	Cervical region extending to the larynx	Day + 3	No	Tisa-cel

## Case 1 – salivary gland localized cytokine release syndrome after anti-CD19 CAR-T therapy

A 72-year-old man was diagnosed with stage IIA diffuse large B-cell lymphoma (DLBCL) presenting with bulky abdominal disease and an International Prognostic Index (IPI) score of 2. His medical history included ischemic heart disease, type 2 diabetes mellitus, and diverticulosis. He initially received six cycles of R-CHOP-based regimen (rituximab, cyclophosphamide, doxorubicin, and prednisone), followed by two additional rituximab infusions and consolidative abdominal radiotherapy due to partial metabolic response at end-of-treatment PET-CT. Subsequent imaging revealed persistent isolated abdominal disease, consistent with primary refractory lymphoma, and the patient was referred for anti-CD19 CAR-T therapy.

Bridging therapy with two cycles of R-GemOx (rituximab, gemcitabine, oxaliplatin) was administered, with stable disease before CAR-T infusion. After standard lymphodepletion with fludarabine and cyclophosphamide, a CD28 costimulated anti-CD19 CAR-T product was infused.

On day + 1 following CAR-T infusion, the patient developed cytokine release syndrome (CRS), graded as Grade 2 according to the American Society for Transplantation and Cellular Therapy (ASTCT) consensus criteria [9], characterized by fever (≥ 38 °C), hypotension requiring fluid support, and low-flow oxygen requirement (nasal cannula at 2 L/min). Tocilizumab was promptly administered, resulting in partial clinical improvement and downgrade to Grade 1 CRS. A second dose of tocilizumab was required on day + 4 because of recurrent hypotension, after which systemic symptoms resolved.

**Fig. 1:**
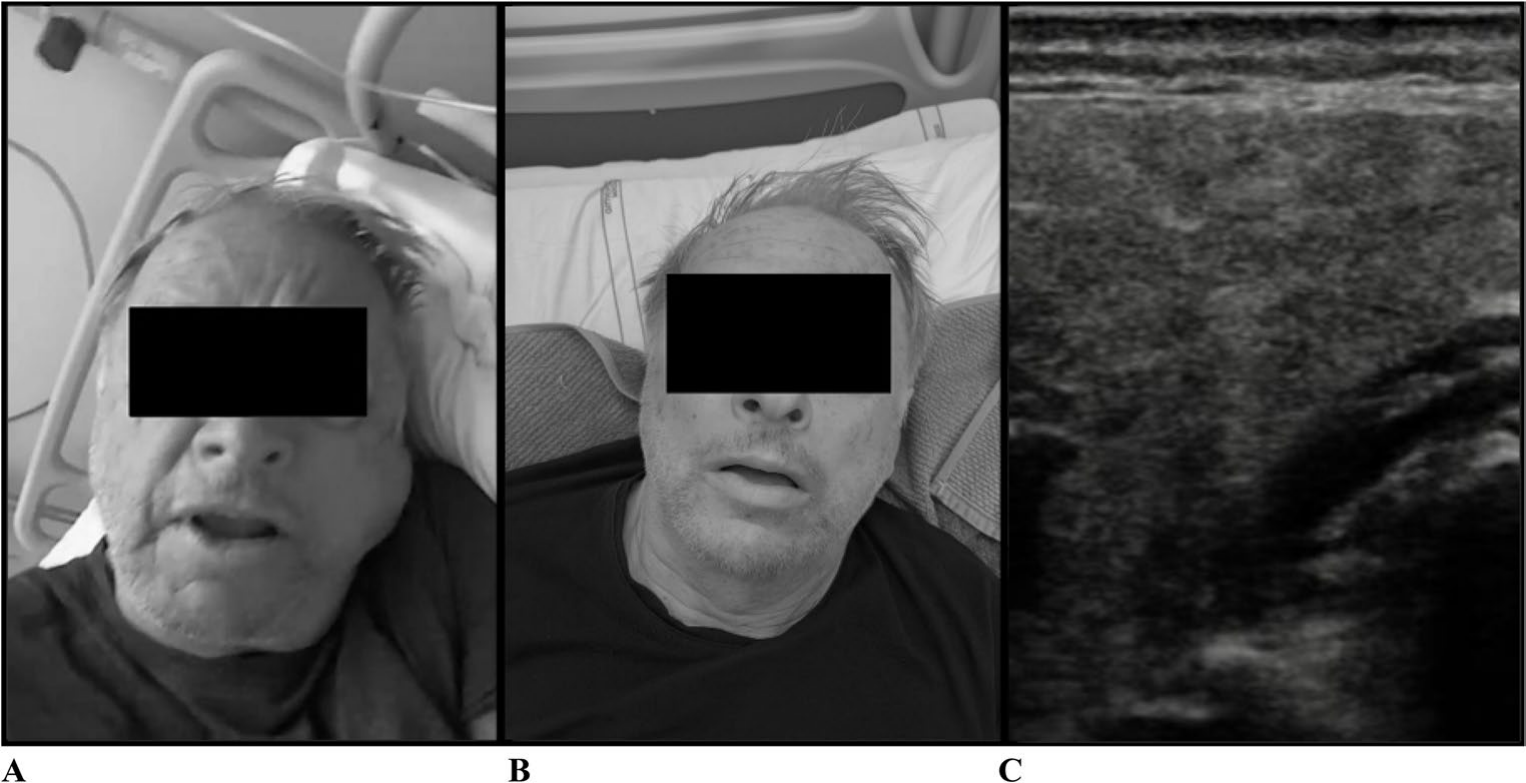
(**A**) Presentation of localized CRS (L-CRS) (**B**) clinical resolution of L-CRS after single bolus of corticosteroid therapy (**C**) mild parotid parenchymal hypoechogenicity on salivary gland ultrasound (B-mode)

On day + 5 post-infusion, the patient developed sudden xerostomia and acute bilateral cervical swelling involving both parotid and submandibular salivary glands, with marked left-sided predominance and local tenderness (Fig. 1A). The rapid increase in glandular volume raised concern for potential airway compromise.

Oropharyngoscopic evaluation excluded mucosal angioedema or visible inflammatory involvement of the oropharynx. Manual compression of the salivary glands demonstrated reduced salivary flow from the left parotid gland, without purulent discharge. Salivary gland ultrasound (Fig. 1C) revealed mildly decreased echogenicity of the left parotid gland, compatible with inflammatory involvement, with no evidence of sialolithiasis or abscess formation.

A comprehensive diagnostic workup was performed to exclude alternative etiologies. Infectious causes were investigated with peripheral and central blood cultures, procalcitonin measurement, chest X-ray, urine culture, and viral serologies (including CMV, EBV, hepatitis viruses, and Toxoplasma), all of which were negative. Autoimmune screening, including antinuclear antibodies (ANA), extractable nuclear antigen antibodies (ENA), and anti-SSA/Ro and anti-SSB/La antibodies, did not support an underlying autoimmune condition. 

**Fig. 2:**
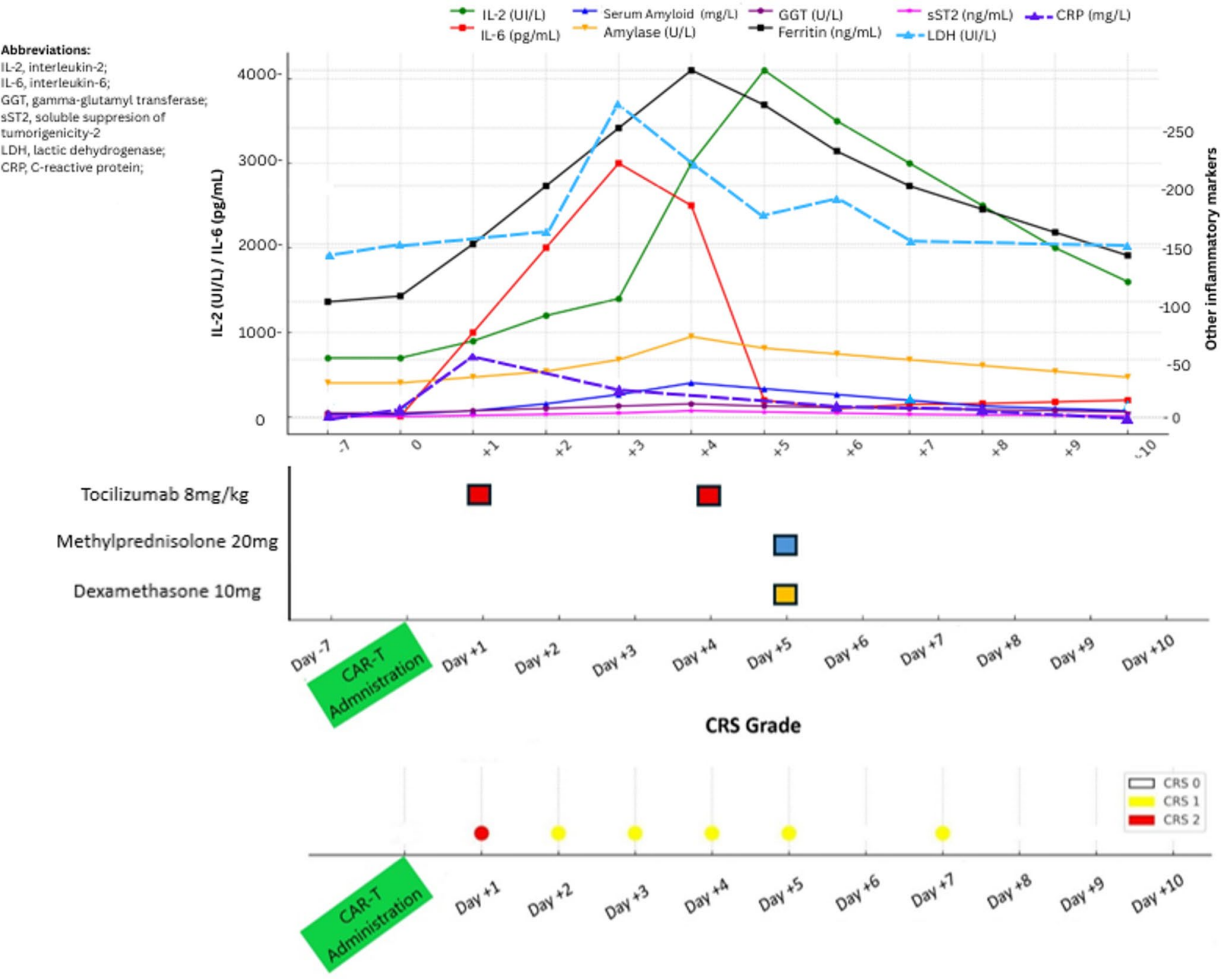
Inflammatory cytokine trends and CRS

Laboratory analysis showed elevated salivary amylase levels, together with increased gamma-glutamyl transferase, soluble ST2, and interleukin-2 receptor concentrations, all of which normalized during follow-up (Fig. 2). Ferritin levels and systemic inflammatory markers did not show a new significant surge suggestive of recurrent systemic CRS.

Importantly, the diagnostic evaluation was performed after the onset of glandular swelling (between day + 5 and day + 6 post-infusion), in parallel with the initial therapeutic interventions.

Given the temporal sequence—systemic CRS followed by abrupt compartmentalized salivary gland inflammation—together with imaging findings and exclusion of alternative etiologies, a diagnosis of localized cytokine release syndrome (L-CRS) was considered. Because of the rapid progression of cervical swelling and concern for airway patency, intravenous methylprednisolone 20 mg was initially administered. In the absence of clinical improvement, treatment was escalated to dexamethasone 10 mg, given its higher anti-inflammatory potency. A prompt clinical response was observed shortly after dexamethasone administration, with marked improvement within a few hours and near-complete resolution of swelling at approximately 6 h (Fig. 1B), although xerostomia persisted transiently for the subsequent three days. Dexamethasone was not continued beyond the initial administration.

The patient experienced no recurrence of cervical swelling and was discharged on day + 10 post-CAR-T infusion in stable clinical condition.

During follow-up, no immune effector cell-associated neurotoxicity syndrome (ICANS) was observed. Response assessment by PET imaging at 30 days post-infusion showed a partial response (Deauville score 4), followed by complete metabolic response (Deauville score 3) at the subsequent evaluation one month later, which was confirmed at 6 months post-CAR-T therapy. Corticosteroid administration did not appear to adversely impact treatment efficacy.

## Case 2 – acute cervical swelling shortly after tocilizumab administration

A 55-year-old woman had been diagnosed at the age of 53 with stage IISA diffuse large B-cell lymphoma (DLBCL), presenting with splenic involvement and subdiaphragmatic lymphadenopathy, with an International Prognostic Index (IPI) score of 1. Her past medical history was notable for nodular thyroid disease, hypertension, diverticulosis, secondary cystic ectasia of the pancreatic duct, and flattening of the sinotubular junction of the ascending aorta.

She received six cycles of first-line R-CHOP chemotherapy followed by two additional doses of rituximab, achieving complete metabolic remission on end-of-treatment ^18F-FDG PET/CT (Deauville score 2). Six months later, imaging revealed a 23-mm pulmonary nodule in the right upper lobe, and histological evaluation confirmed relapse of DLBCL. Given early relapse within 12 months, the patient was referred for treatment with a CD28 costimulated anti-CD19 CAR-T product. At that time, disease involvement was limited to the lungs. A lobectomy was performed for diagnostic purposes, and subsequent PET imaging showed no residual metabolically active disease. Therefore, no bridging therapy was administered prior to CAR-T infusion.

Following lymphodepletion with fludarabine and cyclophosphamide, a CD28 costimulated anti-CD19 CAR-T product was infused. On day + 3 post-infusion, the patient developed cytokine release syndrome (CRS), graded as Grade 1 according to the American Society for Transplantation and Cellular Therapy (ASTCT) consensus criteria [9], characterized by fever (≥ 38 °C) without hypotension or oxygen requirement. Empiric antibiotic therapy was initiated. On day + 5, due to persistent fever lasting more than 48 h and not responsive to antipyretic therapy, first dose of tocilizumab was administered. 

**Fig. 3:**
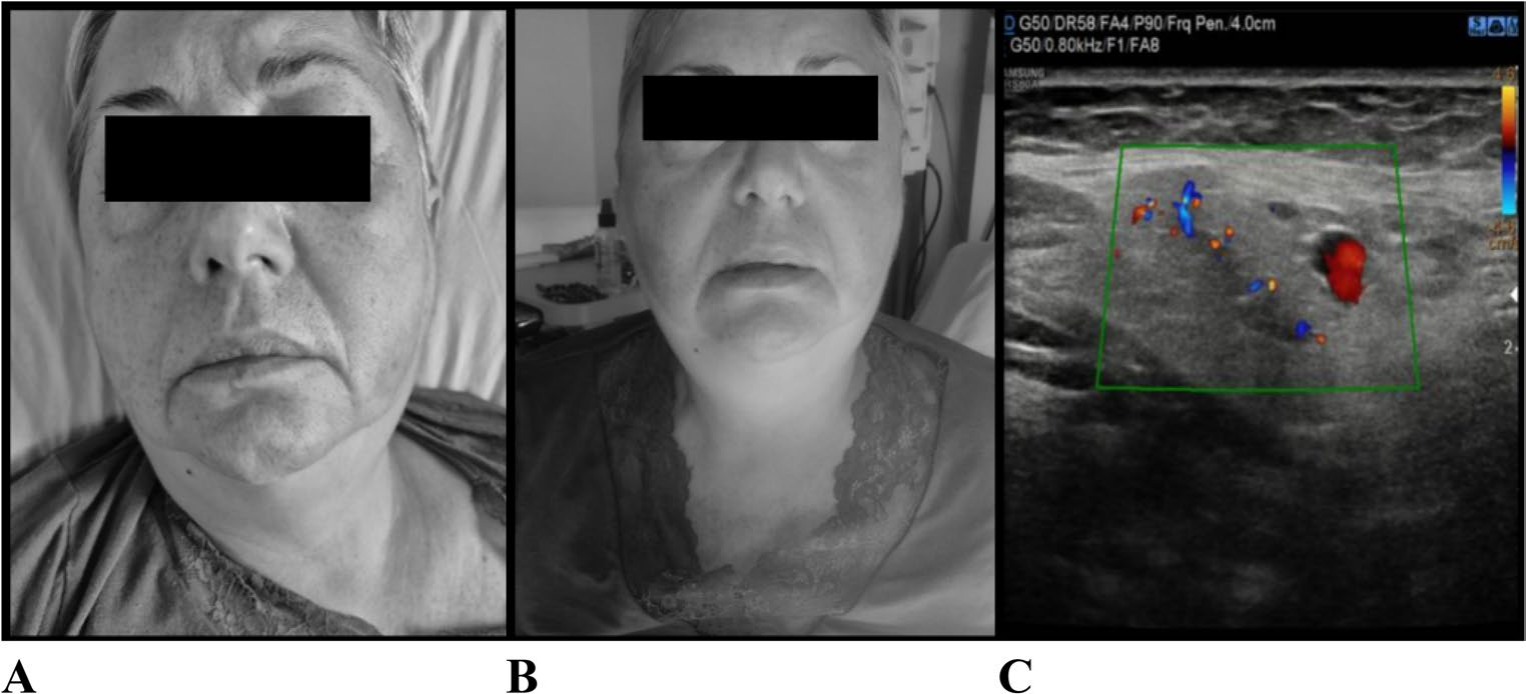
Acute bilateral submandibular swelling (**A**, **B**) developing shortly after tocilizumab administration, in the absence of local inflammatory signs on imaging (**C**)

Within 1–2 h after tocilizumab infusion, the patient developed acute bilateral submandibular swelling associated with subjective dyspnea in the supine position, despite preserved oxygen saturation and stable hemodynamic parameters (Fig. 3A and B). No rash, urticaria, pruritus, or hypotension were observed, and peripheral eosinophil count remained within normal limits. The patient did not report xerostomia. 

A focused diagnostic evaluation was performed to exclude alternative causes. Neck ultrasound did not reveal inflammatory changes of the salivary glands or evidence of ductal obstruction (Fig. 3C). Otolaryngologic assessment excluded upper airway compromise and suggested a possible drug-related hypersensitivity or infusion-related reaction. No clinical or laboratory findings suggestive of infection were identified.

Objective confirmation of a hypersensitivity reaction, such as serum tryptase measurement, was not available; therefore, a definitive causal relationship with tocilizumab cannot be established.

The patient was treated with intravenous chlorphenamine 10 mg and dexamethasone 10 mg, with initial clinical improvement observed within the first few hours and progressive reduction of swelling thereafter, leading to complete resolution within approximately 24 h. Low-flow oxygen was administered overnight as a precaution and discontinued the following day. Dexamethasone was not continued beyond the initial administration.

**Fig. 4:**
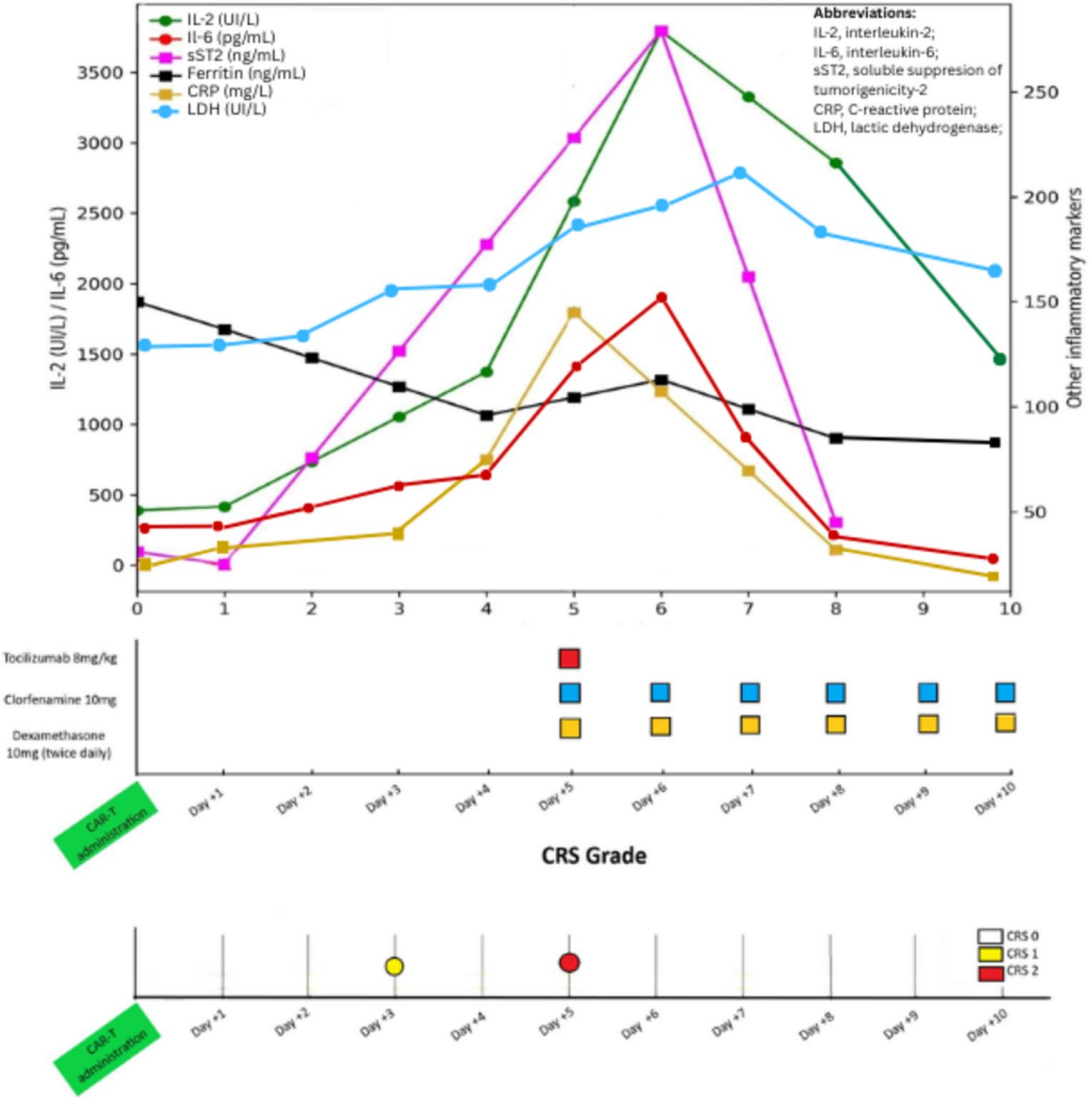
The delayed rise in IL-2 and IL-6 after the dashed vertical line, although less visually prominent for IL-6 due to its more modest increase compared to other biomarkers, in the absence of a concomitant ferritin increase, supports a cytokine pattern more consistent with a hypersensitivity or infusion-related reaction to tocilizumab rather than a localized CRS

Serial laboratory assessments showed a transient increase in IL-6 and IL-2 levels after tocilizumab administration, without a concomitant rise in ferritin (Fig. 4). This cytokine pattern was not suggestive of evolving or compartmentalized CRS, and no additional systemic inflammatory features developed.

The patient experienced complete resolution of symptoms and was discharged on day + 11 post-CAR-T infusion.

During follow-up, no immune effector cell-associated neurotoxicity syndrome (ICANS) was observed. PET imaging at 30 days post-infusion demonstrated complete metabolic response, which was confirmed at 3 and 6 months post-CAR-T therapy. Corticosteroid administration was not associated with an apparent reduction in treatment efficacy.

## Discussion

Localized cytokine release syndrome (L-CRS) involving the craniocervical region represents a heterogeneous clinical entity whose spectrum has progressively expanded in recent reports. While early descriptions focused on tumor-compartment–restricted inflammation, subsequent evidence has demonstrated that L-CRS may occur even in the absence of overt local disease involvement, challenging the traditional “on-target, on-tumor” paradigm.

Wei et al. [5] first proposed a mechanistic model in which CAR-T cell activation within lymphoma-involved compartments may trigger localized inflammatory amplification preceding or accompanying systemic CRS. However, Luan et al. [4] extended this concept by describing L-CRS in systemic B-ALL, suggesting a sequential evolution from systemic CRS (S-CRS) to a redistribution phase characterized by tissue-specific inflammatory localization. This framework supports the notion that L-CRS may represent a compartmentalized hyperinflammatory phase emerging after the initial systemic cytokine surge rather than a strictly tumor-site–restricted phenomenon.

Clinical reports further illustrate the variability of this localized phenotype. Kawase et al. [2] and Inoue et al. [10] described bilateral salivary gland swelling occurring several days after CAR-T infusion in patients without cervical tumor burden, reinforcing the concept of organ-specific inflammation independent of direct malignant infiltration. Ko et al. [11] demonstrated radiological involvement extending beyond the glands to deep cervical soft tissues and the supraglottic region, highlighting that L-CRS may affect anatomically confined spaces with potential airway implications.

More severe presentations were reported by Nakanishi et al. [12] and Shima et al. [13], where laryngeal edema developed early and posed immediate airway risk. Jin et al. [14] and Nakamura et al. [15] described rapidly progressive craniocervical edema requiring urgent airway management in some cases. Importantly, several of these reports noted incomplete control with tocilizumab alone and subsequent need for corticosteroid therapy, underscoring that once tissue-level inflammatory amplification is established, IL-6 receptor blockade may be insufficient.

Across these reports, the onset of localized CRS generally occurred within the first week after CAR-T infusion, most commonly between day 3 and day 5, although later presentations have also been described. In several cases, L-CRS developed within 24–48 h following tocilizumab administration, suggesting a temporal association without establishing a causal relationship.

Taken together, these observations support a model in which L-CRS represents a secondary compartmentalized inflammatory escalation phase characterized by tissue infiltration of activated immune effectors and localized cytokine amplification. The anatomical distribution—from isolated salivary gland involvement to diffuse craniocervical soft-tissue edema—likely reflects variability in tissue susceptibility and local microenvironmental factors rather than differences in systemic cytokine burden alone.

Importantly, this compartmentalized inflammatory escalation may occur despite stabilization or decline of systemic inflammatory parameters, suggesting that once local immune amplification is established, the process may become partially dissociated from circulating cytokine dynamics.

Within this mechanistic framework, the clinical course observed in our first patient—systemic CRS followed by delayed bilateral salivary gland inflammation responsive to corticosteroids—fits closely with previously described salivary gland–predominant L-CRS phenotypes and further supports the concept of organ-specific inflammatory localization after initial systemic activation.

In this context, the salivary gland–predominant presentation observed in our first patient represents a prototypical example of compartmentalized inflammatory escalation, occurring after systemic CRS stabilization and rapidly controlled by corticosteroids. This pattern reinforces the concept that tissue-level inflammatory amplification may become relatively independent from systemic cytokine dynamics once established.

However, not all acute cervical swelling occurring after CAR-T therapy necessarily reflects this compartmentalized inflammatory progression.

In contrast, Case 2 followed a different temporal and biological pattern. Acute bilateral submandibular swelling developed within 1–2 h after tocilizumab administration, in the absence of rash, eosinophilia, or hemodynamic instability, and without imaging evidence of glandular inflammation. Moreover, ferritin levels did not increase, and no progression of systemic inflammatory features occurred. Although IL-6 levels rose transiently after tocilizumab infusion, this phenomenon is a well-recognized pharmacodynamic effect of IL-6 receptor blockade, resulting from reduced receptor-mediated clearance rather than true inflammatory amplification.

While tocilizumab is widely used and generally well tolerated in the management of CRS, acute infusion-related and hypersensitivity reactions have been reported in non-hematologic settings.Baenziger-Sieber et al. reported oropharyngeal swelling occurring shortly after tocilizumab infusion, mimicking angioedema, in the absence of systemic inflammatory features [6]. Park et al. described immediate anaphylactic reactions to tocilizumab in patients with inflammatory diseases [7]. More broadly, immediate and delayed hypersensitivity reactions to biologic agents, including IL-6 receptor inhibitors, have been reviewed in the literature [8].

Although our second case lacked classical features of IgE-mediated anaphylaxis, the close temporal association with tocilizumab infusion and rapid response to antihistamine and corticosteroids raise the possibility of an infusion-related or non–IgE-mediated hypersensitivity reaction. Importantly, such a presentation may clinically mimic L-CRS in the post–CAR-T setting, particularly when swelling involves the cervical region.

Whenever rapid biopsy may not be feasible, inflammatory cytokine dynamics, local inflammatory symptoms such as xerostomia, imaging, and timing of tocilizumab, may drive the correct diagnosis towards L-CRS vs. drug reaction; in patients with autoimmune diseases, the organ involvement may also help in identifying LICATS.

In this differential framework, imaging may provide clinically relevant support: ultrasound can rapidly identify salivary gland enlargement, altered echogenicity, ductal obstruction, or abscess formation, whereas the absence of inflammatory glandular changes may favor an alternative diagnosis such as an infusion-related reaction rather than salivary gland–predominant L-CRS, as suggested by previously reported cases [11, 12].

The distinction between L-CRS and drug-related reactions is not merely academic. L-CRS reflects tissue-level inflammatory activation within the broader spectrum of CAR-T toxicity and may require immunosuppressive escalation to prevent airway compromise. In contrast, infusion-related reactions may not indicate ongoing CAR-T–driven inflammation and may resolve rapidly with supportive therapy.

## Conclusions

Salivary gland involvement with xerostomia represents a genuine and clinically significant manifestation of L-CRS, which may require intense exposition to corticosteroids to be resolvered; nevertheless, not all acute cervical swelling immediately after CAR-T therapy should be automatically attributed to L-CRS, and differential diagnosis with an adverse reaction to tocilizumab, LICATS, anaphylaxis, or pseudoflare need to be carried out in order to tailor the acute management.

## Data Availability

No datasets were generated or analysed during the current study.
